# Supplementary material for: Transcriptomic profiling implicates PAF1 in both active and repressive immune regulatory networks
Source: BMC Genomics. 2022 Nov 30;23:787. doi: 10.1186/s12864-022-09013-6 (PMC9713194; doi:10.1186/s12864-022-09013-6)
Supplement: Supplementary file 1 — Additional file 1: S1 Figure. Immune stress marginally affects PAF1 nuclear localization. (A) Immunofluorescence microscopy shows how selected immune stimuli do not significantly disrupt the nuclear localization of PAF1. A549 cells were stimulated for 3 hours with the given stimuli before being fixed and then stained with PAF1 antibody (red) and Hoechst (blue) for marking nuclei. Images were produced with epifluorescence microscopy. All scale bars represent 50 μM. (B) Microscopy images were assessed quantitatively for PAF1 nuclear localization. Cell nuclei were selected for with StarDist, using Hoechst staining as the training control. Grey mean area was used to measure intensity of PAF1 signal in these nuclei (as represented by each point), relative to the average of the total grey mean area for each image. These values were plotted as a distribution. Statistical significance was determined using an unpaired two-tailed Student’s t-test. S2 Figure. Further characterization of immune activation by stimuli. (A) PCA shows distinct gene expression profile of stimulations relative to mock across both ncgRNA and PAF1 KO cell line. The counts for all genes were corrected with a variance-stabilizing transformation via DESeq2, and only the variability of biological replicates was removed as a batch effect with ComBat. PCA was performed on the resulting counts, with the top 2 principal components being plotted. (B) Volcano plots show activation of the immune response in ncgRNA cell line following each stimulation. Comparisons were performed within the DESeq2 pipeline. The resulting log2 foldchanges and –log10 p-values are plotted for all genes, where top immune response genes are labeled. Cutoffs are set at log2 foldchange > 1.5 or < − 1.5, and p-value < 0.05. (C) qRT-PCR analysis validates activation of the immune response in ncgRNA and PAF1 KO cells. Fold changes for ISG15 and CCL20 were calculated using the ΔΔCt method for three technical replicates, being normalized again [file 12864_2022_9013_MOESM1_ESM.docx]

**Supplementary Material**

**
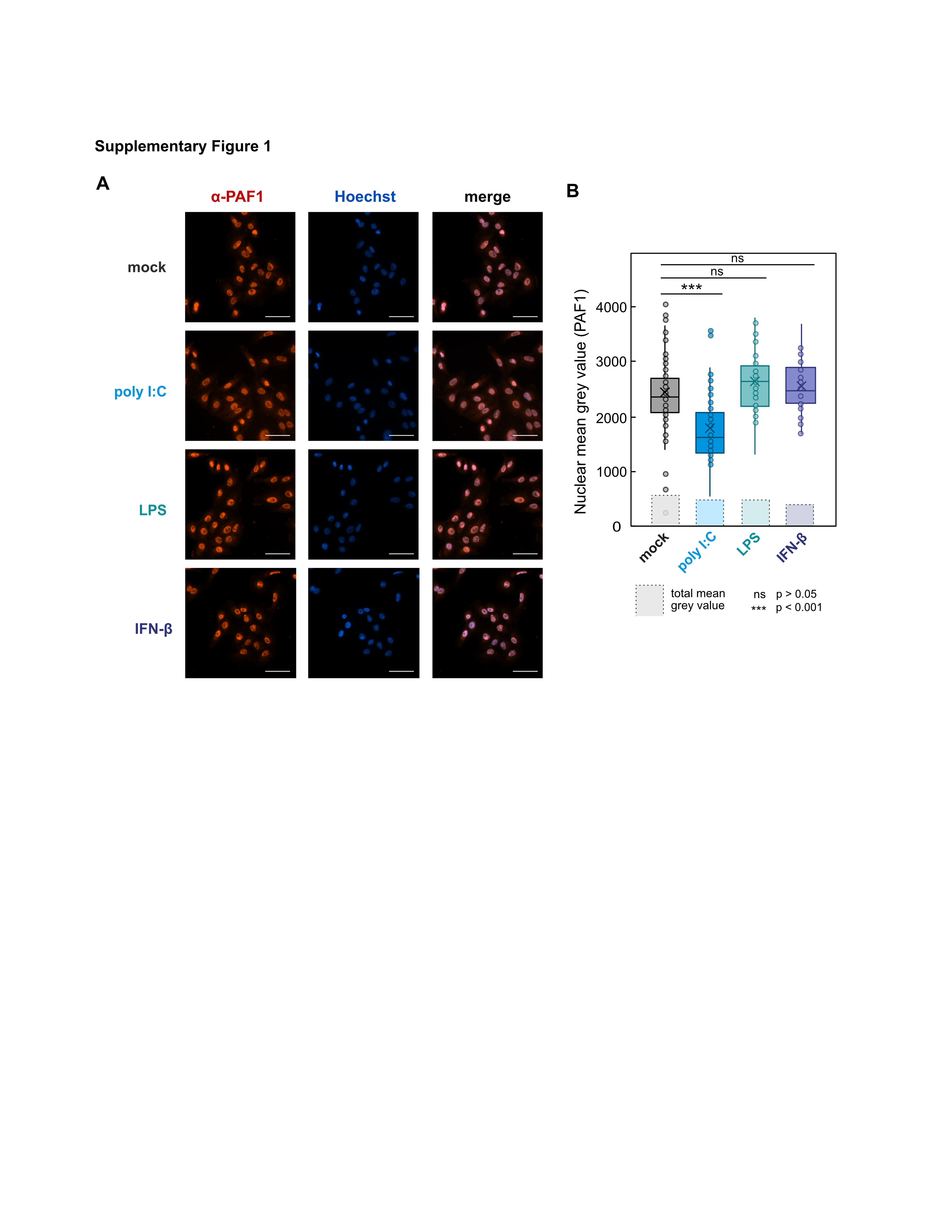
**

**S1 Figure. Immune stress marginally affects PAF1 nuclear localization.**

(A) Immunofluorescence microscopy shows how selected immune stimuli do not significantly disrupt the nuclear localization of PAF1. A549 cells were stimulated for 3 hours with the given stimuli before being fixed and then stained with PAF1 antibody (red) and Hoechst (blue) for marking nuclei. Images were produced with epifluorescence microscopy. All scale bars represent 50 µM. (B) Microscopy images were assessed quantitatively for PAF1 nuclear localization. Cell nuclei were selected for with StarDist, using Hoechst staining as the training control. Grey mean area was used to measure intensity of PAF1 signal in these nuclei (as represented by each point), relative to the average of the total grey mean area for each image. These values were plotted as a distribution. Statistical significance was determined using an unpaired two-tailed Student’s t-test.

**
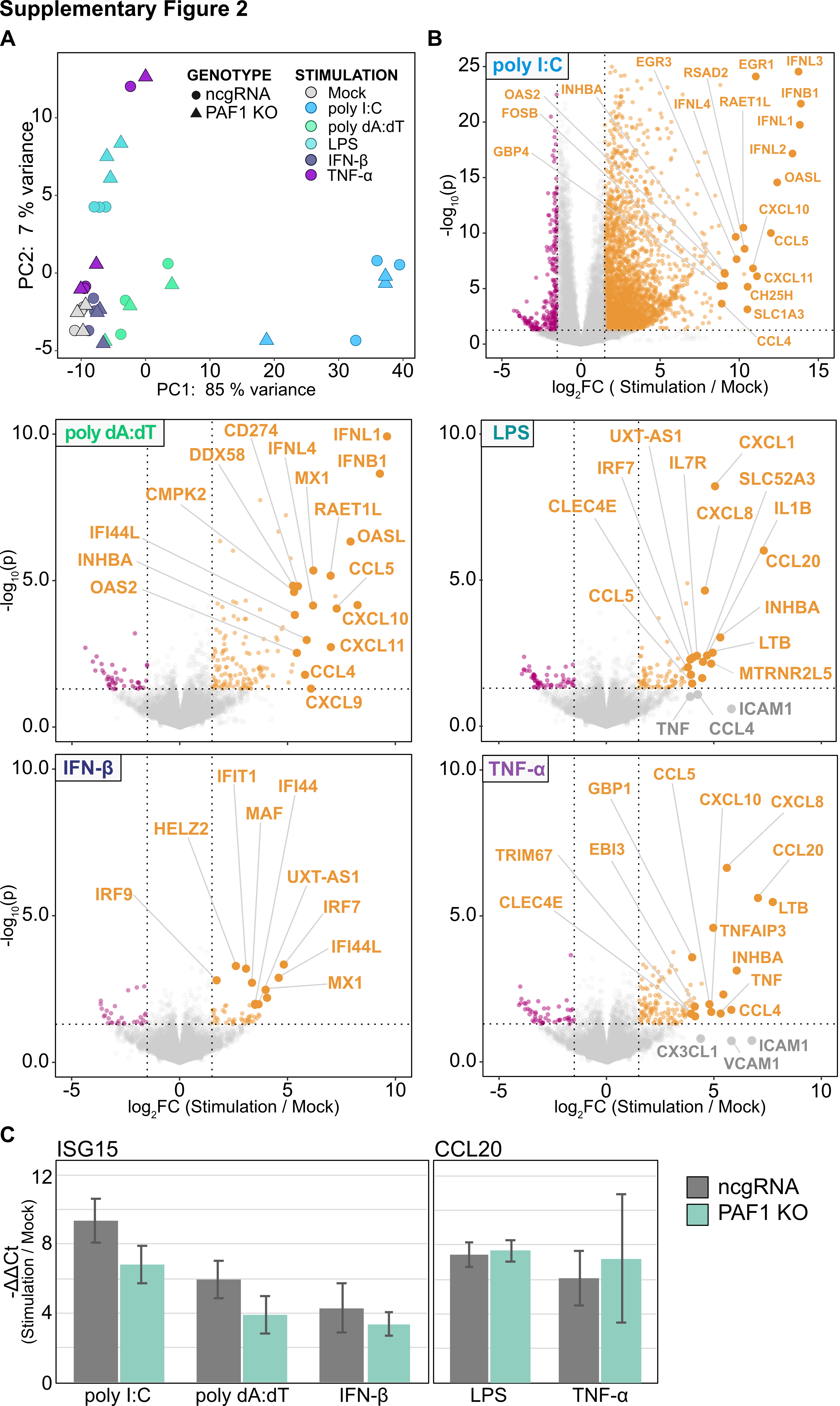
**

**S2 Figure. Further characterization of immune activation by stimuli.**

(A) PCA shows distinct gene expression profile of stimulations relative to mock across both ncgRNA and PAF1 KO cell line. The counts for all genes were corrected with a variance-stabilizing transformation via DESeq2, and only the variability of biological replicates was removed as a batch effect with ComBat. PCA was performed on the resulting counts, with the top 2 principal components being plotted. (B) Volcano plots show activation of the immune response in ncgRNA cell line following each stimulation. Comparisons were performed within the DESeq2 pipeline. The resulting log2 foldchanges and –log10 p-values are plotted for all genes, where top immune response genes are labeled. Cutoffs are set at log2 foldchange > 1.5 or < -1.5, and p-value < 0.05. (C) qRT-PCR analysis validates activation of the immune response in ncgRNA and PAF1 KO cells. Fold changes for ISG15 and CCL20 were calculated using the ΔΔCt method for three technical replicates, being normalized against GAPDH expression. Error bars indicate standard deviation across three biological replicates.

**
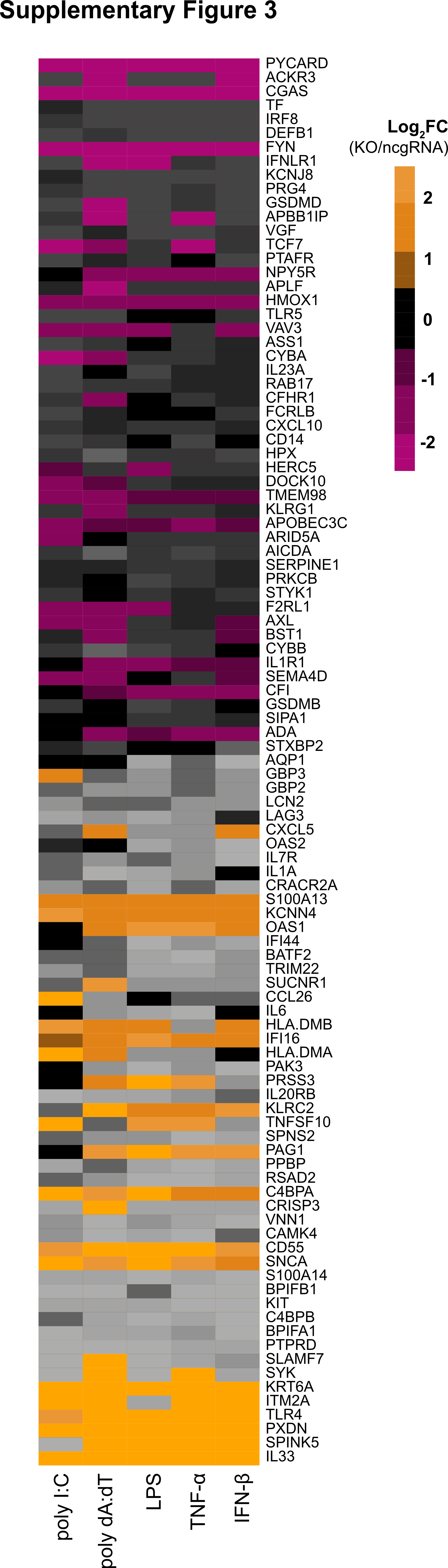
**

**S3 Figure. Top PAF1-dependent genes across stimuli.**

Heatmap shows relative changes in gene expression as log2 foldchanges of DEGs from PAF1 KO versus ncgRNA contrasts of Figure 2. Colors are desaturated for genes with an adjusted p-value > 0.05.


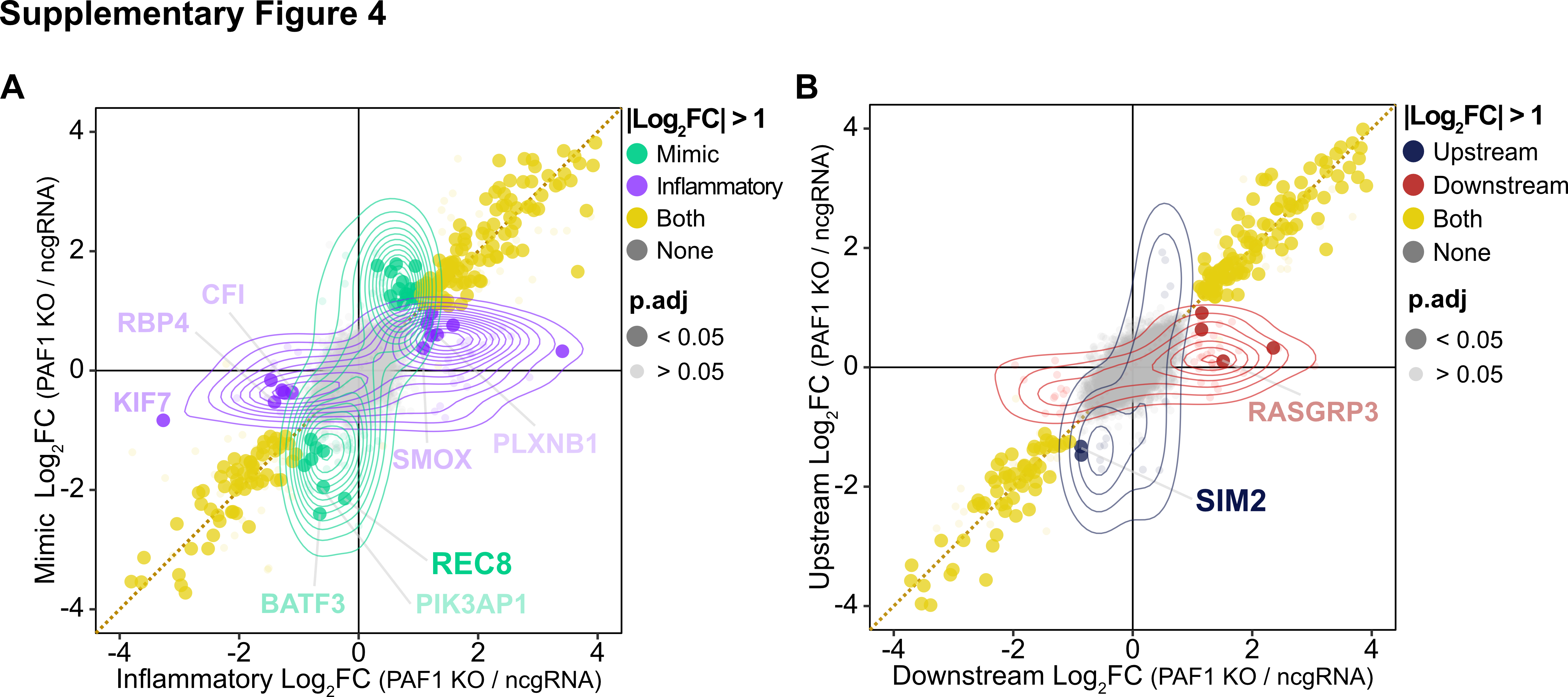


**S4 Figure. PAF1 alters gene expression unique to some immune stimuli.**

Dot plots show PAF1-dependent genes include variability across the type of stimulation. (A) The log2 foldchanges and adjusted p-values for PAF1 KO versus ncgRNA cells were averaged across interferon (poly I:C, poly dA:dT) and inflammatory (LPS, TNF-α) stimuli. The average log2 foldchanges were plotted on two axes. Density plots are shown for all genes with log2 foldchanges greater than 1 or less than -1. Significant genes (p.adj < 0.05) are plotted as larger, bolded points and notable genes are labeled. Genes with strong induction (avg. log2 fold-change > 1) in ncgRNA cells are bolded. (B) The same analytical approach discussed above is applied to PAF1 KO DEGs for upstream (poly I:C, poly dA:dT, LPS) versus downstream (TNF-α, IFN-β) stimuli.


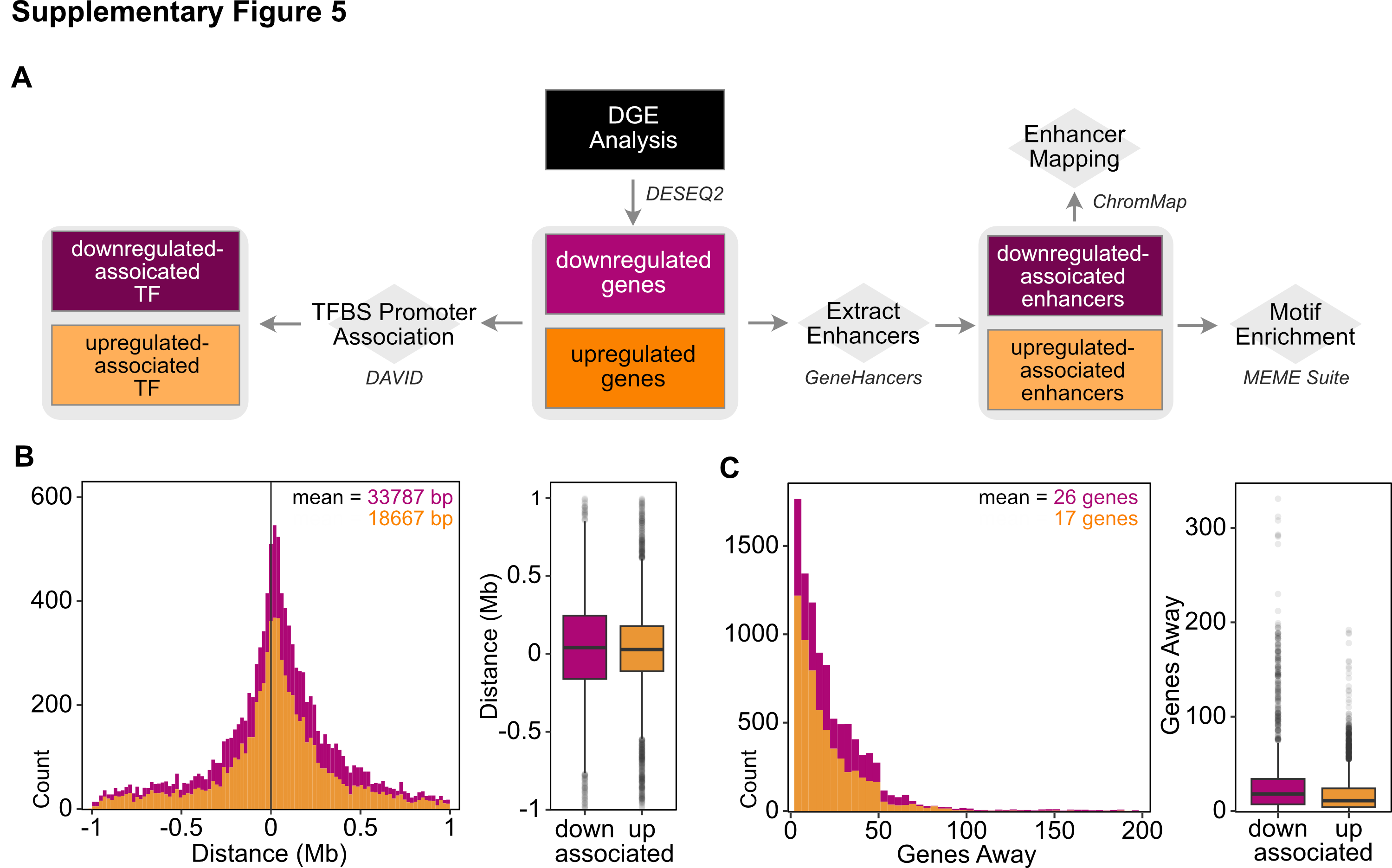


**S5 Figure. PAF1-associated enhancer analysis pipeline and quality control.**

(A) Summarization of pipeline for promoter and enhancer motif analyses. (B) Distribution of distances of enhancer motifs from target upregulated (orange) or downregulated (magenta) gene. (C) Distribution of the number of genes in between enhancer motif and target upregulated (orange) or downregulated (magenta) gene.

**

**

**S6 Figure. Transcription factor and predicted targets network.** Identified enhancer motifs associated with known transcription factors map to immune genes (GO:0002376). Significantly enriched enhancer motifs (E < 0.05, enrichment ratio > 2) were screened against the TRRUST v2 database for target genes and the type of interaction. Non-mapping motifs include those that were either not transcription factors or had no known associations in the database. Target genes with consistent differential expression across stimuli for PAF1 KO versus ncgRNA (average log2 foldchange > 1 or < -1) were colored accordingly (red = downregulated, blue = upregulated, grey = did not meet criteria or insufficient reads).

**Table S1. DESeq2 outputs.**

**Table S2. GSEA outputs.**

**Table S3. Associated promoter and enhancer motifs.**

**Table S4. Known regulon analysis.**

**Table S5. qRT-PCR primers.**

**Table S6. Host-dependency factors.**
